# Inter-center variability in the application of the Lake Louise Criteria to the diagnosis of myocarditis

**DOI:** 10.1186/1532-429X-18-S1-P249

**Published:** 2016-01-27

**Authors:** Yoko Mikami, Tiago Teixeira, Johannes Schüler, Jeanette Schulz-Menger, Matthias G Friedrich, Peter Bernhardt, Sophie Mavrogeni, Leslie T Cooper, Carmen P Lydell, Andrew G Howarth

**Affiliations:** 1 grid.22072.350000000419367697Stephenson Cardiac Imaging Centre, Libin Cardiovascular Institute of Alberta, University of Calgary, Calgary, AB Canada; 2 grid.14848.310000000122923357Montreal Heart Institute, Montreal, QC Canada; 3 grid.6363.00000000122184662Charité Universitätsmedizin Berlin and HELIOS-Clinics, Berlin, Germany; 4 grid.63984.300000000090644811McGill University Health Centre and RI-MUHC, Montreal, QC Canada; 5 grid.6582.90000000419369748University of Ulm, Ulm, Germany; 6 grid.419873.00000000406227521Onassis Cardiac Surgery Center, Athens, Greece; 7 grid.417467.70000000404439942Mayo Clinic, Jacksonville, FL USA; 8 grid.6363.00000000122184662Charité Universitätsmedizin Berlin, Berlin, Germany

## Background

An international Consensus Group established the proposed cardiovascular MR (CMR) criteria for diagnosis of acute myocarditis (Lake Louise Criteria) in 2009, consisting of 3 tissue markers: irreversible tissue injury on late gadolinium enhanced (LGE) images, edema on T2 STIR images and hyperemia on early enhanced (EE) images. The purpose of this study was to test the variability between the different CMR centers for the diagnosis of myocarditis based on Lake Louise Criteria.

## Methods

CMR images of 20 patients (18 male, mean age 38 years) without known pre-existing cardiovascular disease with suspected acute myocarditis based on symptoms and clinical findings were analyzed. Fourteen patients of 20 were reported myocarditis positive by Lake Louise Criteria in the clinical read. LGE images, T2-STIR images and EE images were evaluated at 2 separate CMR centers by experienced readers at the respective corelabs. The patients were categorized as Lake Louise Criteria positive if 2 of the following 3 markers are positive: visual presence of non ischemic pattern focal LGE, visual presence of tissue edema and/or T2 ratio ≥ 2.0 on T2-STIR images, EE ratio ≥ 4.0 on EE images. The agreement of each marker and Lake Louise Criteria between 2 sites was evaluated.

## Results

The number of patients positive for each imaging marker at site 1 and site 2 respectively were: LGE 18 (90%), 15 (75%); T2-STIR 17 (85%), 12(60%); EE 12 (60%), 15 (75%). The agreement between 2 sites respectively were: LGE 17/20 (85%); T2 11/20 (55%); EE 17/20 (85%). Lake Louise Criteria was positive for 18 patients (90%) at site 1 and 15 patients (75%) at site 2. The agreement of Lake Louise Criteria between 2 sites was 17/20 (85%, kappa = 0.5).

## Conclusions

Except for T2-weighted images, there was good agreement between two experienced centres when using the Lake Louise Criteria for the assessment of patients with suspected myocarditis. T1 mapping may be considered as an alternative to T2-weighted CMR and thus should be tested for inter-center variability in clinical settings.

